# Stability of the Octameric Structure Affects Plasminogen-Binding Capacity of Streptococcal Enolase

**DOI:** 10.1371/journal.pone.0121764

**Published:** 2015-03-25

**Authors:** Amanda J. Cork, Daniel J. Ericsson, Ruby H. P. Law, Lachlan W. Casey, Eugene Valkov, Carlo Bertozzi, Anna Stamp, Blagojce Jovcevski, J. Andrew Aquilina, James C. Whisstock, Mark J. Walker, Bostjan Kobe

**Affiliations:** 1 School of Chemistry and Molecular Biosciences and Institute for Molecular Bioscience, University of Queensland, Brisbane, QLD, 4072, Australia; 2 Australian Infectious Disease Research Centre, University of Queensland, Brisbane, QLD, 4072, Australia; 3 Department of Biochemistry and Molecular Biology and the ARC Centre of Excellence in Structural and Functional Microbial Genomics, Monash University, Melbourne, VIC, 3800, Australia; 4 School of Biological Sciences and Illawarra Health and Medical Research, University of Wollongong, Wollongong, NSW, 2522, Australia; Centers for Disease Control & Prevention, UNITED STATES

## Abstract

Group A *Streptococcus* (GAS) is a human pathogen that has the potential to cause invasive disease by binding and activating human plasmin(ogen). Streptococcal surface enolase (SEN) is an octameric α-enolase that is localized at the GAS cell surface. In addition to its glycolytic role inside the cell, SEN functions as a receptor for plasmin(ogen) on the bacterial surface, but the understanding of the molecular basis of plasmin(ogen) binding is limited. In this study, we determined the crystal and solution structures of GAS SEN and characterized the increased plasminogen binding by two SEN mutants. The plasminogen binding ability of SEN^K312A^ and SEN^K362A^ is ~2- and ~3.4-fold greater than for the wild-type protein. A combination of thermal stability assays, native mass spectrometry and X-ray crystallography approaches shows that increased plasminogen binding ability correlates with decreased stability of the octamer. We propose that decreased stability of the octameric structure facilitates the access of plasmin(ogen) to its binding sites, leading to more efficient plasmin(ogen) binding and activation.

## Introduction

*Streptococcus pyogenes* (group A *Streptococcus*, GAS) is a human pathogen that causes a wide array of infections, ranging from uncomplicated episodes to serious invasive diseases and post-infection sequelae. Globally, the disease burden of GAS infection is high, with over 700 million non-invasive episodes and almost two million severe infections occurring each year [1,2]. Thus, it is of interest to understand the interaction between this pathogen and the human host. The cell surface of GAS harbors a spectrum of virulence factors, some of which are capable of interacting with host proteins [3,4]. These interactions may serve as a vehicle for the initiation and progression of GAS infection. One host protein implicated in streptococcal infection is the human serine protease plasminogen. Plasminogen, once converted to its active form plasmin, can degrade extracellular matrix components through its trypsin-like proteolytic activity, thus participating in the breakdown of tissue barriers [5]. Plasminogen that is bound and activated by GAS contributes to migration and proliferation of the bacteria into areas of the body that are normally sterile. GAS can acquire plasminogen directly via cell-surface plasminogen receptors, or indirectly via fibrinogen receptors. These receptors are capable of binding a trimolecular complex consisting of plasminogen, streptokinase and fibrinogen [6]. There are four known receptors present on the surface of GAS that can bind plasminogen directly. These are the streptococcal surface enolase (SEN) [7], glyceraldehyde-3-phosphate dehydrogenase (GAPDH; also known as streptococcal plasmin receptor, Plr, or streptococcal surface dehydrogenase, SDH) [8,9], plasminogen-binding group A streptococcal M-like protein (PAM) [10], and the PAM related protein (Prp) [11]. These proteins are known to interact with lysine binding sites (LBS) within the kringle domains (KR) 1, 2, 4 and 5 of plasminogen [6]. Although SEN has the ability to bind plasmin(ogen), it also catalyzes the conversion of 2-phosphoglycerate to phosphoenolpyruvate as part of the glycolytic pathway inside the cell [7,12].

Alpha-enolases are typically homodimeric, however they have been found in some bacterial species including GAS to be octameric, consisting of a tetramer of dimers [13–15]. The primary plasminogen-binding site (binding site 1, BS1) has been proposed to correspond to the segment of SEN containing the C-terminal lysine [16]. Recent studies have identified an additional binding site (binding site 2, BS2) that comprises the internal loop containing lysines 252 and 255 [17]. It was shown that site-directed mutations targeting the lysine residues of these binding sites abolished plasminogen binding, while the structural integrity of the octamers and the glycolytic activity of the enzymes were retained. Interestingly, the structure of SEN from *S*. *pneumoniae* showed that BS1 was buried in the subunit interface and not readily accessible [15].

To characterize the plasminogen-binding sites in SEN, we have previously targeted for mutagenesis several lysine residues in the C-terminal region of the protein [17], where lysine residues with roles in plasminogen binding had earlier been identified [7,16,18,19]. Unexpectedly, the SEN^K312A^ and SEN^K362A^ mutants showed a 2- and 3.4-fold increase in plasminogen binding, respectively. SEN^K362A^ was also able to activate plasminogen more effectively in the presence of tissue-plasminogen activator (tPA) than the wild-type protein. To understand the molecular basis of this effect, we performed a structural and biophysical analysis of the wild-type and mutant proteins. Their crystal structures reveal an octameric arrangement, consistent with other bacterial enolases [13,15], but unveil an inverse correlation between the size of inter-subunit interfaces and plasminogen binding. SEN^K362A^ also showed a decreased thermal stability. We propose that weaker inter-subunit interactions result in a less rigid octamer with improved access by plasminogen. Furthermore, this study suggests that the binding of SEN introduces changes to plasminogen structure that make it more susceptible to activation by tPA.

## Materials and Methods

### Construction, expression and purification of SEN mutants

The pET14bSEN expression vector [16] was used to construct site-directed mutants as previously described [17], using primers listed in Table 1. DNA sequence analysis was used to confirm introduced mutations using primers given in Table 1. pET14bSEN and SEN mutant constructs were transformed into electro-competent *E*. *coli* BL21 STAR (DE3) cells and expressed and purified as previously described [17].

**Table 1:** Forward primers used for PCR construction and DNA sequence analysis of SEN site-directed mutants.

Introduced mutation	Forward mutagenesis primer
Lys^312^/Ala	5’- ACTGAACGCCTAGGCGCTCGTGTTCAATTGGTT -3’
Lys^362^/Ala	5’- GCTATCGAAATGGCTGCTGAAGCTGGATATACTGCC -3’
Primer	DNA sequence analysis primer
SENmutseq	5’-GGTGACGAAGGTGGATTTGC-3’

### Surface plasmon resonance analysis of plasminogen binding

The circulating form of human plasminogen (Glu-plasminogen) was purified from human plasma using lysine Sepharose 4B affinity chromatography as described previously [20]. The binding of recombinant wild-type and mutant SEN proteins to Glu-plasminogen was measured using a BIAcore® 3000 optical biosensor (GE Healthcare). Glu-plasminogen was immobilized onto a CM5 sensor chip as previously described [17] to yield 10,000 response units. A reference surface was prepared in the absence of Glu-plasminogen in the same manner to account for non-specific binding to the sensor chip. Wild-type SEN and mutant proteins were injected for 500 s at room temperature in 10 mM HEPES, pH 7.4, 0.15 M NaCl, 3 mM EDTA, 0.005% v/v surfactant P20, using a flow rate of 30 μl/min. Proteins were allowed to dissociate for 600 s, followed by regeneration of the surface with two 10-s injections of 10 mM glycine-HCl, pH 1.7 at 100 μl/min. Binding was assayed in quadruplicate, on two independently prepared sensor chips; similar relative binding responses for wild-type and mutant SENs were consistently observed. Kinetic models could not suitably fit the data using the BIAevaluation 3.1 software, likely due to mass transport rate limitations, so the relative affinities of SEN variants for plasminogen binding were only assessed qualitatively.

### ELISA analysis of plasminogen binding

Binding of wild-type and mutant SEN to immobilized plasminogen was also assessed by ELISA as previously described [21], with the following modifications. A microtitre plate was coated with serial dilutions of Glu-plasminogen in 32 mM Na_2_CO_3_.H_2_O, 68 mM NaHCO_3_, pH 9.6, and after the blocking step, wells were incubated with 5 μg of wild-type and mutant SEN. The reactions were developed using SIGMA*FAST^TM^* OPD solution (Sigma-Aldrich) according to manufacturer’s recommendations, and were read at 450 nm using a Spectromax® Plus^380^ microplate reader (Molecular Devices).

### Plasminogen activation assay

Plasminogen prepared from pooled human plasma as previously described [22] was pre-incubated with wild-type or mutant SEN at 37°C for 10 min. The rate of plasminogen activation was measured in triplicate in a final volume of 200 μL/reaction at 37°C for 3–4 hours. Each reaction contained 15 nM plasminogen, 100 μg/mL SEN, 0.15 mM chromogenic substrate S-2251 (Chromogenix) in a reaction buffer consisting of 50 mM Tris pH 7.4, 100 mM NaCl, 0.01% v/v Tween-80. The reaction was initiated by the addition of 0.5 mg/mL of tPA (Actilyse, Boehringer Ingelheim) and the progress of plasminogen activation was monitored at 405 nm using FLUOstar OPTIMA (BMG Labtech). The initial velocity of plasminogen activation was calculated from the slope of the plots of A_405_ vs. reaction time squared [23].

### Enzymatic (α-enolase) activity

The catalytic activity of the SEN mutants was compared to the wild-type protein by measuring the conversion of 2-phosphoglycerate to phosphoenolpyruvate. The reaction was performed according to the method described by Derbise *et al*. [16].

### Far-UV circular dichroism (CD) spectroscopy

CD spectra were obtained on a JASCO J-810 CD spectropolarimeter at room temperature as described previously [24]. Spectra representing the average of 16 scans from 250 to 190 nm were measured and all spectra were corrected for the baseline by subtracting control spectra of buffer alone. The molar ellipticity per residue was calculated as [mol deg x 100]/[no. of amino acids x pathlength (cm) x peptide concentration (moles/L)].

### Thermal stability assays

Thermofluor assays were set up in triplicate with final concentrations of 20 μM protein, 12.5x Sypro Orange dye (Invitrogen Molecular Probes), 20 mM HEPES pH 7.4, and 150 mM NaCl. Emission spectra was collected between 20 and 95°C on an Applied Biosystems ABI7900HT real-time PCR unit. Results were analyzed by applying Savitzky-Golay smoothing of the raw data and extracting the melting temperature from the peak of the first derivative.

### Size-exclusion chromatography and mass spectrometry

The purified SEN mutants were re-chromatographed using a Superdex-200 10/300 GL column (GE Healthcare) equilibrated in 200 mM NH_4_OAc (pH 6.8) at a flow rate of 0.4 ml/min. The eluent was collected at one-minute intervals with the three most strongly absorbing fractions across each peak pooled for subsequent mass spectrometry analysis on a Synapt HDMS (Waters) instrument. Instrument conditions were similar to those previously described [17], with the exception of the following voltages: capillary, 1.65 kV; sample cone, 160 V, “trap collision energy,” 25 V; “transfer collision energy,” 15 V. Instrument backing pressure was adjusted to 4.5 mbar to provide sufficient collisional cooling to preserve non-covalent interactions and maintain the proteins in their native conformation. All spectra were externally calibrated using a cesium iodide spectrum and reference file and processed using MassLynx software.

### X-ray crystallography of wild-type and mutant SEN

Wild-type and mutant SENs were crystallized from 1–3.5 M sodium/potassium phosphate buffer (pH 5–7.5) containing 2% v/v PEG 400 by vapor diffusion using the hanging-drop method. The mutant protein crystals were isomorphous with those of the wild-type protein and had the symmetry of the space group P4. Diffraction data ranging from 2.1–2.9 Å resolution was collected at 100 K and a wavelength of 0.9795 Å on the MX2 beamline at the Australian Synchrotron (Melbourne, Australia) using Blu-Ice software [25], and processed using XDS [26] and SCALA [27]. Structures were solved by molecular replacement using Phaser [28] and the *S*. *pneumoniae* α-enolase structure (PDB entry 1w6t) [15] as a template. Automated model building and refinement of SEN mutants was performed in PHENIX [29] using Autobuild and Refine, respectively. Refinement of wild-type SEN generated more interpretable electron density maps when refined using REFMAC5 [30] within the CCP4 suite [30], using twin refinement due to its high twin fraction (0.455 with operator K, H, -L). Iterative model building between refinement rounds was carried out in Coot [31]. The MolProbity [32] Ramachandran plot distribution for the wild-type, SEN^K312A^, and SEN^K362A^ structures places 94.59, 96.76, and 97.33 percent residues in the favored region, and 0.24, 0.29, and 0.52 percent as outliers, respectively. The crystallographic data statistics are summarized in Table 2. The structures have been deposited in the Protein Data Bank (PDB ID 3ZLH [wild-type enolase], 3ZLF [the K312A mutant] and 3ZLG [the K362A mutant]).

**Table 2:** Crystallographic data collection and refinement statistics.

	Wild-type	SEN^K312A^	SEN^K362A^
**Data collection**			
Space group	*P*4	*P*4	*P*4
Cell dimensions			
*a*, *b*, *c* (Å)	185.8, 185.8, 56.3	181.6, 181.6, 56.4	187.3, 187.3, 57.2
α, β, γ (°)	90, 90, 90	90, 90, 90	90, 90, 90
Resolution (Å)	19.75–2.90 (3.00–2.90)^*a*^	90.81–2.15 (2.23–2.15) ^*a*^	19.97–2.1 (2.17–2.10) ^*a*^
*R*_meas_	0.432 (1.127)	0.241 (1.206)	0.149 (1.5)
<*I* / σ(*I*)>	4.60 (1.91)	10.64 (2.13)	14.16 (2.10)
Completeness (%)	99.69 (98.83)	99.99 (100.00)	100.00 (100.00)
Multiplicity	6.0 (5.0)	11.8 (10.1)	11.3 (7.1)
Twin fraction	0.460	0.134	0.109
**Refinement**			
Resolution (Å)	2.9	2.15	2.10
No. unique reflections	43076 (4208)	100879 (10040)	116292 (11538)
*R*_work_ / *R*_free_	0.153 / 0.205	0.173 / 0.199	0.179 / 0.207
No. atoms			
Protein	13172	13919	13704
Ligand/ion	0	40	40
Water	0	577	424
Average *B*-factors			
Protein	52.87	40.06	39.41
Ligand/ion	-	47.06	64.58
Water	-	30.51	33.64
R.m.s. deviations			
Bond lengths (Å)	0.016	0.005	0.006
Bond angles (°)	1.730	0.866	0.926

^*a*^ Each dataset was collected from a single crystal; the values in parentheses are for the highest-resolution shell. *R*_meas_ = *∑*_*hkl*_(*N*(*hkl*)/[*N*(*hkl*)-1])^1/2^ ∑_*i*_|I_*i*_(*hkl*)- <*I*(*hkl*)>|/ ∑_*hkl*_∑_*i*_I_*i*_(*hkl*), where *I*_*i*_(*hkl*) is the intensity of the *i*th measurement of an equivalent reflection with indices *hkl*.

### Small-angle X-ray scattering

Purified wild-type SEN was dialyzed for 18 h into 20 mM HEPES, pH 7.4, 150 mM NaCl, 1 mM DTT, and a concentration series from 6.4 to 1.5 mg/mL was prepared. Small-angle X-ray scattering data was collected at the SAXS/WAXS beamline of the Australian Synchrotron (Melbourne, Australia), using a PILATUS 1M detector at a distance of 1.6 m and wavelength of 1.12713 Å, which yielded a range of momentum transfer 0.011 < *q* < 0.500 Å^-1^, where *q* = 4π.sin(*θ*)/*λ* and 2*θ* is the scattering angle. A 50 μL sample volume was exposed while flowing through a 1.5 mm diameter quartz capillary at 298 K, at a rate of 1 μL/s. Groups of 1-s images were scrutinized for variation, and consistent images were normalized to transmitted intensity, averaged, buffer-subtracted and converted to absolute scale against the scattering cross-section of water using ScatterBrain (http://www.synchrotron.org.au/index.php/aussyncbeamlines/saxswaxs/software-saxswaxs). Further processing was performed using the ATSAS 2.5 software package [33–35]. The forward scatter *I*(0) and radius of gyration *R*_g_ were determined via Guinier analysis for *q*.*R*_g_ < 1.3 using AUTORG. These were also calculated from *P*(*r*) distributions obtained by indirect transformation with AUTOGNOM. Volumes were calculated using AUTOPOROD, and molecular weights were estimated using SAXS MoW [36]. Datasets were examined for concentration dependence and linearity in the Guinier region, and the 1.5 mg/mL data was used for modeling. *Ab initio* modeling was conducted using the bead-modeling program DAMMIF [37] in the ATSAS Online suite [35]. Twenty independent reconstructions under four-fold rotational symmetry were generated from the distance distribution obtained from data with the *q*-range limited to 0.01 < *q <* 0.15 Å^-1^. These reconstructions were then clustered into groups based on normalized spatial discrepancy using DAMCLUST [34,38,39], yielding averaged spread regions for each cluster. Additionally, GASBOR [40] was used to generate a dummy residue reconstruction incorporating higher-*q* data. This used a *P*(*r*) function obtained from data with the *q*-range limited to 0.01 < *q <* 0.31 Å^-1^, and was also restrained by four-fold rotational symmetry.

### Protein docking analysis of SEN and plasminogen

Rigid body docking was performed with the crystal structures of human plasminogen (PDB entry 4dur) [22] and SEN^K312A^ (this study). The SEN^K312A^ mutant was chosen over the wild-type protein because its biophysical behavior is closest to the wild-type while being a higher resolution and a more complete structure. Given the flexible nature of plasminogen, the protomer was broken up into discrete kringle domains and docked separately. Each kringle domain was then docked using the PatchDock server [41], with geometric constraints applied to SEN to avoid its regions involved in forming the inward-facing surface of the octameric ring. Each group of one hundred docking solutions generated per kringle domain were further refined and rescored with the FireDock server [42,43]. The ten top-scoring docking solutions for every kringle domain were superimposed with the complete plasminogen structure, and evaluated based on clashes and plausibility.

## Results

### X-ray crystal structure of wild-type GAS SEN

**Fig 1:**
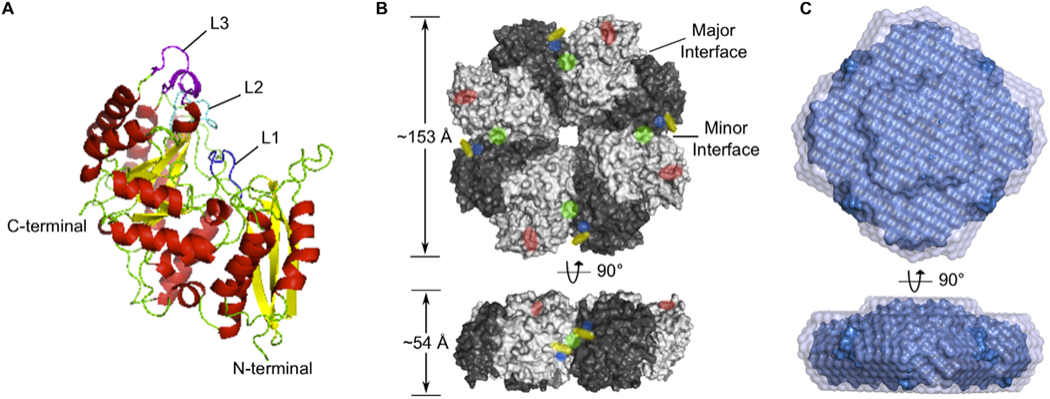
Structure of streptococcal α-enolase. (**A**) Structure of a monomeric unit of SEN. The three loop regions L1, L2 and L3 are colored blue, cyan and magenta, respectively. The elements of secondary structure are colored red (α-helices) and yellow (β-sheets). (**B**) Structure of SEN octamer. The major and minor interfaces are labeled. BS1 and BS2 are colored blue and red, respectively; and lysine residues mutated in this study are colored yellow (K312) and green (K362). (**C**) Results of *ab initio* modeling from SAXS data. A dummy residue model is shown in solid blue within the averaged spread region, shown in transparent purple, for a similar cluster of bead models.

To provide the foundation for understanding the structural basis of the virulence function of GAS SEN, we determined its crystal structure at 2.9-Å resolution (Table 2). The structure was solved by molecular replacement using the α-enolase structure from *S*. *pneumoniae* (93% sequence identity to α-enolase from *S*. *pyogenes*; PDB entry 1w6t) as a search model [15]. The asymmetric unit of the crystal contains two dimers, with each subunit exhibiting a kidney shape. Each subunit is made up of an N-terminal domain (residues 1–143) and a C-terminal domain (residues 144–435). The N-terminal domain consists of three anti-parallel β-strands, followed by four α-helices that are connected by the flexible loop L1 (residues 38–45). The C-terminal domain contains the widely occurring 8-stranded β/α barrel architecture [44,45]. The barrel, however, does not contain the typical (βα)_8_ fold as found in triosephosphate isomerase (TIM)-barrel enzymes [46], but rather comprises a β_2_α_2_(βα)_6_ fold that is also seen in enolases from yeast or other bacterial origins [15,47,48]. The C-terminal region also contains two loop regions L2 (residues 152–159) and L3 (residues 245–266), which together with L1 close the active site once enolase binds the substrate (Fig. 1A). Plasminogen BS1 is located at the C-terminus, while BS2 is located on the catalytic loop L2.

**Fig 2:**
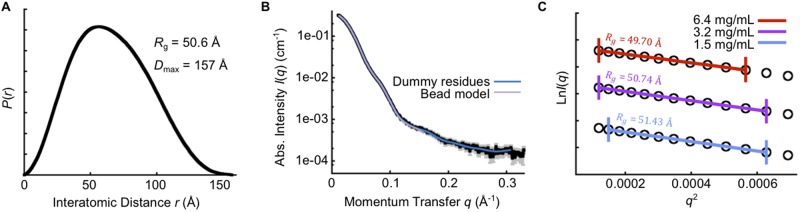
Small-angle X-ray scattering (SAXS) data. (**A**) Pairwise interatomic distance distribution derived from SAXS. The maximum distance (*D*_max_) from the *P*(*r*) is 157 Å, and the radius of gyration (*R*_g_) is 50.6 Å. (**B**) Experimental scattering of wild-type SEN on absolute, logarithmic scale, with 1*σ* error bars shown in gray. Theoretical fits of the *ab initio* models (Fig. 1C) are shown in blue for the dummy residue model, and purple for the cluster-representative bead model. (**C**) Guinier transformation of the data from a concentration series, demonstrating concentration-dependent *R*_g_ variation of < 5% and linearity over the region *q*.*R*_g_ < 1.3.

In solution, GAS SEN exists as an octamer [17]. In the crystals, the octamer is made up of a tetramer of homo-dimers and consists of two types of interfaces. Dimerization of two enolase molecules involves the ‘major interface’, whereas octamerization occurs through the ‘minor interface’ (Fig. 1B). Each major and minor interface buries a total surface area of 3648 Å^2^ and 2580 Å^2^, respectively (calculated by PISA [49]), with the octamer having an approximate diameter of 153 Å and thickness of 54 Å. A particle of this size is also consistent with small-angle X-ray scattering (SAXS) data, from which we identified a maximum particle dimension of 157 Å (Fig. 2A), and a volume-derived molecular weight of 369.4 kDa. Furthermore, SAXS-based *ab initio* modeling restrained by four-fold rotational symmetry was able to restore shapes consistent with the crystal structure (Fig. 1C and Fig. 2B). BS1 is located in the minor inter-subunit interface.

### Plasminogen-binding and activation ability of SEN and its mutants

**Fig 3:**
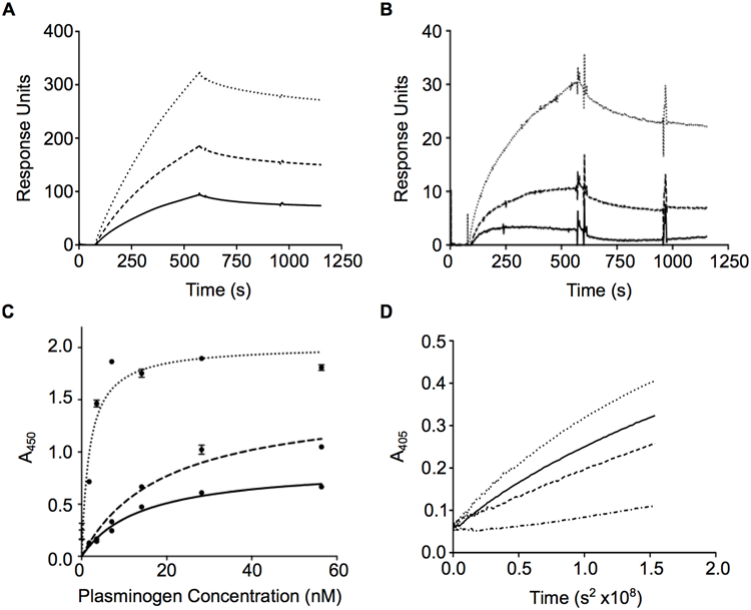
Plasminogen binding and activation of wild-type and mutant SEN. (**A**) Surface plasmon resonance sensorgrams for 100 nM recombinant wild-type SEN (solid), SEN^K312A^ (dashed) and SEN^K362A^ (dotted) binding to immobilized human Glu-plasminogen (~10,000 RUs). Binding experiments were performed in quadruplicate and the curves represent the average. (**B**) Surface plasmon resonance sensorgrams using a lower level of immobilised human Glu-plasminogen (~1,000 RUs). Analysis was carried out according to *(A)* and curves follow the same key. (**C**) ELISA analysis for the binding of wild-type SEN (solid), SEN^K312A^ (dashed) and SEN^K362A^ (dotted) (5 μg/mL) to immobilized Glu-plasminogen. The graph shown was constructed from two independent experiments with samples in duplicate, and error bars indicate the standard deviation. (**D**) tPA activation of plasminogen in the absence (dot-dash) and presence of recombinant wild-type SEN (solid), SEN^K312A^ (dashed) and SEN^K362A^ (dotted) at 100 μg/mL. Plasminogen activation experiments were performed in triplicate and the curves represent the average.

Site-directed mutagenesis was undertaken previously to examine the role of several lysine residues within the C-terminal region of SEN in plasminogen-binding [17]. The degree of binding was measured qualitatively using surface plasmon resonance. Wild-type SEN binding to immobilized human Glu-plasminogen occurred at a slow rate, and the dissociation was similarly slow and did not go to completion. This behavior is not unexpected for a large octameric molecule and is largely due to mass transport limitations, whereby global fitting of kinetic models can yield arbitrary rate constants due to changes in net reaction fluxes at the sensor chip surface [50]. Surprisingly, two SEN mutants bound more plasminogen than the wild-type protein, with the percent increase in plasminogen binding for SEN^K312A^ and SEN^K362A^ corresponding to 196% and 341%, respectively (Fig. 3A). The binding responses for the mutants remain in the same order when using a lower-density surface (Fig. 3B). Wild-type SEN and the mutants all bound plasminogen in a dose-dependent manner, as shown by ELISA (Fig. 3C); the apparent equilibrium constants (K_D_ ± standard error), calculated assuming one-site specific binding, corresponded to 14.11 ± 2.28 nM, 19.32 ± 4.82 nM and 1.853 ± 0.337 nM for the wild-type, SEN^K312A^ and SEN^K362A^ proteins, respectively. The analysis of plasminogen-binding abilities among the SEN variants is consistent between the ELISA and surface plasmon resonance approaches.

We further examined the effects of wild-type and mutant SEN on plasminogen activation by tPA. Plasminogen was mixed with the SEN proteins and activation was initiated with the addition of tPA. The amidolytic activity of the plasmin formed in this way was monitored spectrophotometrically by measuring absorption at 405 nm. In the presence of wild-type SEN, the initial rate of tPA-dependent plasminogen activation significantly increased by more than 20-fold when compared with the no-SEN control (Fig. 3D). Interestingly, the activation rate of SEN^K362A^ (3.73 x 10^-9^ OD/s^2^) increased while SEN^K312A^ (1.237 x 10^-9^ OD/s^2^) decreased, to 1.75 and 0.58-fold, of the wild-type activity (2.123 x 10^-9^ OD/s^2^), respectively. This observation suggests that parameters other than binding must contribute to the plasminogen-activating ability of SEN.

### Enolase activity of SEN mutants

**Fig 4:**
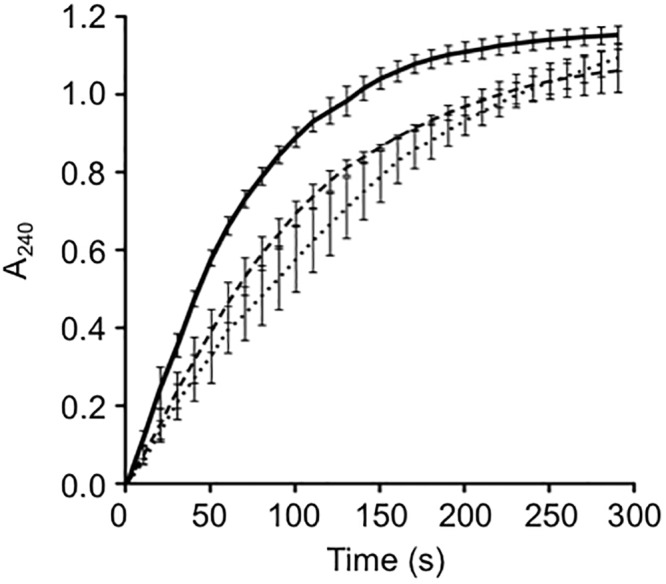
Enzymatic activity of recombinant wild-type SEN and mutant forms. The graph shown was constructed from the averages of three independent experiments, with error bars indicating the standard deviation: wild-type SEN (solid), SEN^K312A^ (dashed) and SEN^K362A^ (dotted).

The glycolytic activity of recombinant wild-type SEN, *i*.*e*. the conversion of 2-phosphoglycerate to phosphoenolpyruvate, was measured spectrophotometrically by measuring absorption at 240 nm. The amount of phosphoenolpyruvate produced over a time period of 5 min was recorded and plotted (Fig. 4). The initial rate of phosphoenolpyruvate formation was calculated to be 131 nmol/min/μg of recombinant protein. Enzymatic activity was reduced by approximately 38% and 46% for SEN^K312A^ (81.2 nmol/min/μg) and SEN^K362A^ (71.21 nmol/min/μg), respectively. Enolase activity has no known role in plasminogen binding and activation.

### Biophysical characterization of wild-type and mutant SEN

**Fig 5:**
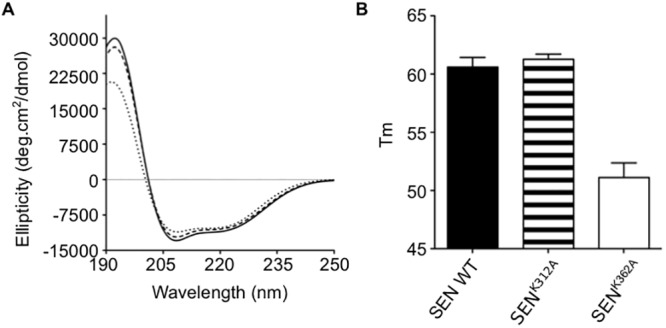
Biophysical characterization of wild-type and mutant SEN. (**A**) Far-UV circular dichroism spectra demonstrating the predominantly α-helical secondary structure of recombinant wild-type SEN (solid), SEN^K312A^ (dashed) and SEN^K362A^ (dotted). (**B**) Comparison of the thermal stability of wild-type SEN (black), SEN^K312A^ (striped) and SEN^K362A^ (clear) determined by the thermofluor assay. The assay was performed in triplicate and error bars indicate the standard deviation.

**Fig 6:**
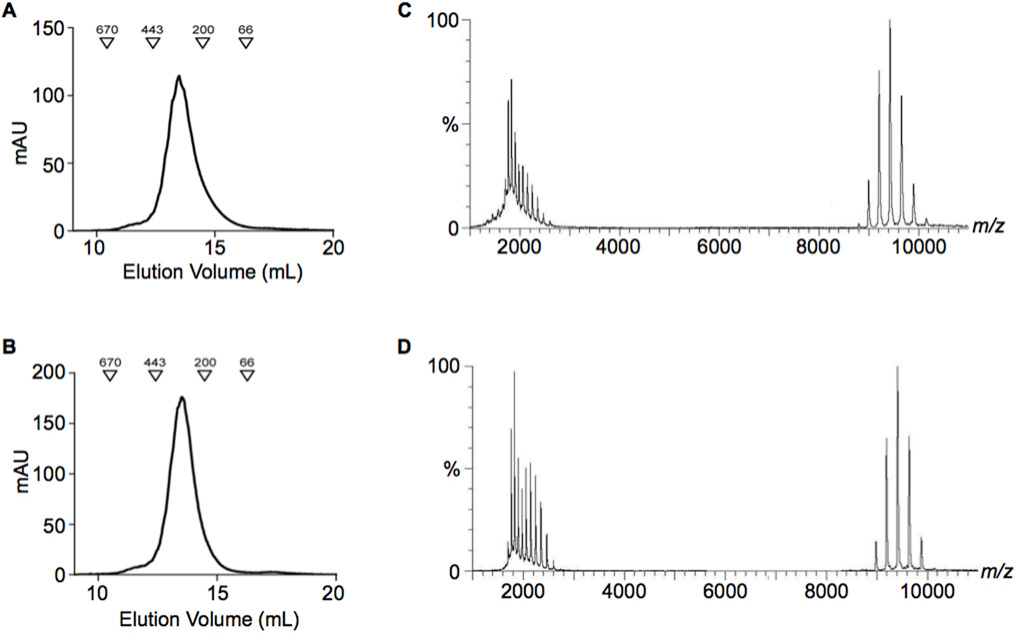
Size-exclusion chromatography and mass spectrometry analysis of wild-type SEN and SEN^K312A^. Size-exclusion chromatography elution profile of (**A**) wild-type SEN and (**B**) SEN^K312A^ shows that both proteins elute as a single mono-disperse peak. Molecular weight standards are indicated by triangles. Subsequent mass spectra of (**C**) wild-type SEN and (**D**) SEN^K312A^ illustrate that both proteins exist as octamers.

**Fig 7:**
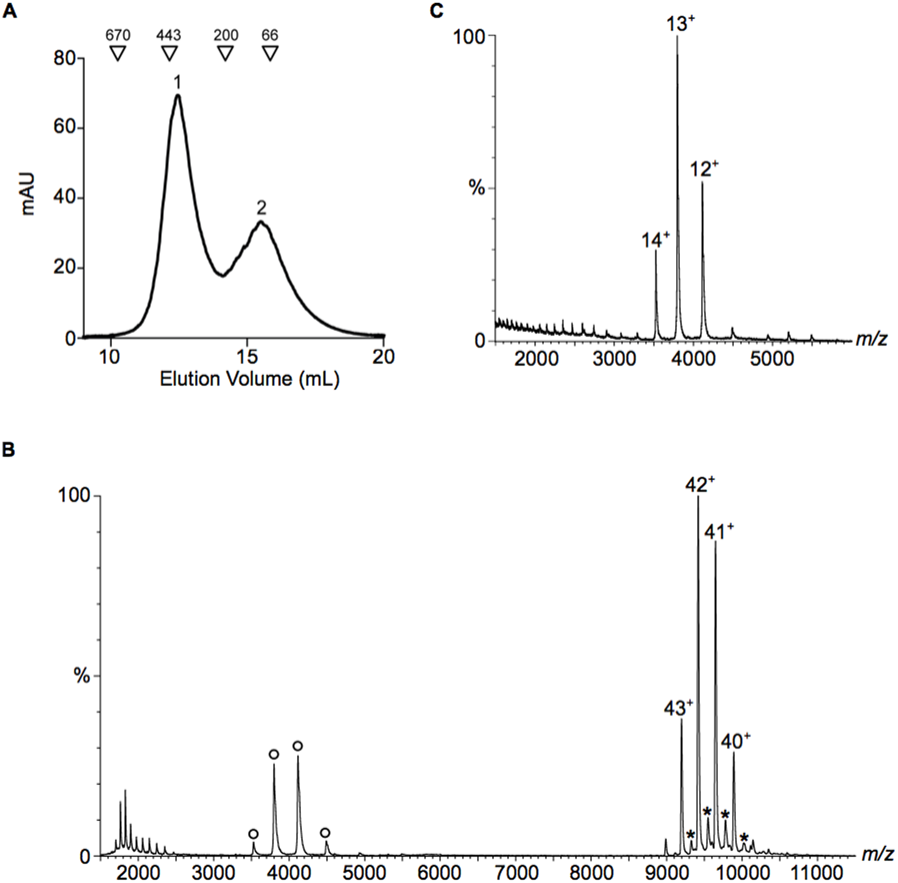
Size-exclusion chromatography and native mass spectrometry analysis of SEN^K362A^. (**A**) Size-exclusion chromatography elution profile showing that the protein elutes as two peaks. Molecular weight standards are indicated by triangles. (**B**) Mass spectrum illustrating that elution peak 1 is composed of a mixture of octameric, heptameric (*) and monomeric (o) species. (**C**) Mass spectrum showing that elution peak 2 contains monomeric species only.

Biophysical experiments were carried out to determine if the increase in plasminogen binding of the SEN mutants can be attributed to the substitution of an interacting residue or if the mutation affects the structural integrity of the protein. Both SEN^K312A^ and SEN^K362A^ were found to be correctly folded and to retain the α-helical secondary structure of the wild-type protein, as deduced from circular dichroism (CD) spectra, which showed a positive band at ~195 nm and negative bands at ~209 and 222 nm (Fig. 5A). Differential scanning fluorimetry (thermofluor) experiments showed that the thermostability of SEN^K312A^ was similar to the wild-type protein, with the melting point of SEN^K312A^ and wild-type SEN corresponding to 61.3°C and 60.6°C, respectively (Fig. 5B). The melting point of SEN^K362A^ however, was reduced to 51.1°C, indicating that it was less stable than the wild-type protein. Size-exclusion chromatography in preparation for mass spectrometry showed that while wild-type SEN and SEN^K312A^ eluted as a single peak (Fig. 6A and Fig. 6B), SEN^K362A^ eluted as two peaks (Fig. 7A). This is consistent with the mass spectrum of SEN^K362A^ acquired under conditions where non-covalent interactions are preserved, which demonstrates that there is a mixture of monomer, heptamer and octamer present in the first peak to elute, indicative of a solution state equilibrium between these species (Fig. 7B). The mass spectrum of the second peak however corresponded to monomeric SEN^K362A^ only (Fig. 7C). By contrast, for wild-type SEN and SEN^K312A^, only intact octamers could be detected (Fig. 6C and Fig. 6D).

### X-ray crystal structures of SEN^K312A^ and SEN^K362A^

**Fig 8:**
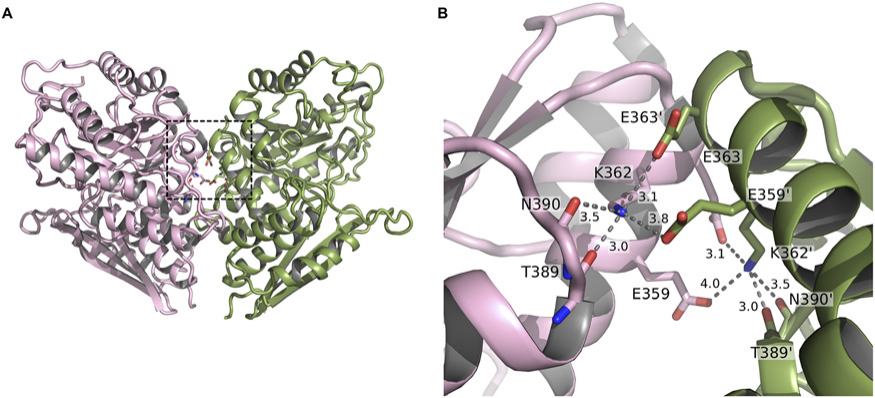
K362 interactions in wild-type SEN. (**A**) SEN minor interface with residues interacting with K362 shown as stick representations. (**B**) Close-up view of the area bounded by a dashed line in *(A)* showing the potential hydrogen bond network involving K362, T389 and N390 of the same monomer, and E359’ and E363’ of the opposing monomer.

The crystal structures for SEN^K312A^ and SEN^K362A^ were determined at 2.15 Å and 2.10 Å resolution, respectively (Table 2), and were consistent with the wild-type protein in terms of octameric structure and packing of secondary structural elements. There were, however, differences in the subunit interface area when compared to the wild-type protein, with the total buried surface area for SEN^K312A^ and SEN^K362A^ corresponding to 24,230 Å^2^ and 23,070 Å^2^, respectively (the corresponding value for wild-type SEN is 24,912 Å^2^). A trend of an inverse relationship between plasminogen binding and the total interface area can be deduced. K362 forms several interactions in the minor interface that may function to stabilize the octamer. Each K362 is within hydrogen-bonding distance of up to four other entities: carbonyl oxygens of T389 and N390 in a nearby loop on the same protomer, and side chains of E359’ and E363’ from the opposing protomer (Fig. 8). By contrast, K312 is not directly involved in inter-subunit contacts.

### Molecular docking and proposed mechanism of plasminogen binding by SEN

**Fig 9:**
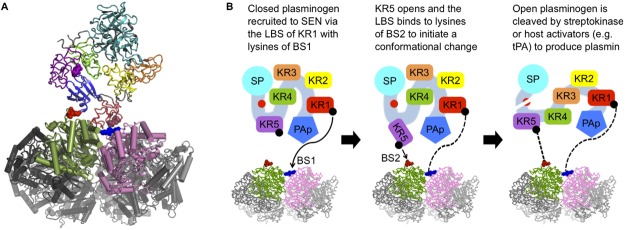
Molecular docking results and mechanism of plasminogen binding by SEN. (**A**) Human plasminogen KR1 docked at the SEN minor interface. Plasminogen domains are colored as follows: pan-apple (PAp) domain blue; KR1 red; KR2 yellow; KR3 orange; KR4 green; KR5 purple; serine protease (SP) domain cyan. Linear three-residue patches on KR1 and KR5 implicated in lysine binding are shown as spheres. One SEN dimer is colored green and pink, respectively, with the remaining octamer colored in shades of grey. One occurrence of SEN BS1 and BS2 is shown as blue and red spheres, respectively. (**B**) Proposed model for the binding of plasminogen by SEN involving interactions between BS1 and KR1, and BS2 and KR5. The color scheme follows *(A)*. Figure adapted from Law *et al*. [22].

Rigid-body docking analysis suggests that plasminogen kringle 1 (KR1) may be involved in direct interaction with SEN. A top-ten scoring docking solution places the LBS of KR1 in close proximity of BS1 in the SEN minor interface (Fig. 9A). Furthermore, this docking solution places the LBS on KR5 in a position to potentially interact with SEN BS2 without being sterically hindered. A previous structural study has demonstrated that in closed plasminogen, KR5 is able to exist in two distinct conformations—a fully closed state, and a second conformer where KR5 has peeled away from the plasminogen core, thus partially exposing its LBS [22]. This mobility is suggested to be key for triggering the opening of plasminogen [22]. Based on all the available data, we are thus able to propose a mechanism of SEN binding to plasminogen (Fig. 9B). Upon binding of plasminogen KR1 to SEN BS1, we suggest that KR5 becomes accessible to BS2, and once bound initiates a conformational change of plasminogen that gives rise to its cleavage to produce plasmin by plasminogen activators. Additionally, some of the top-scoring solutions from the docking calculations also indicate that the potential interface area may not be large and potentially one copy of plasminogen may be bound by each subunit of the SEN octamer. This may lead to cooperative effects and increased avidity, counteracting the low affinities of individual molecules when higher stoichiometric ratios are considered.

## Discussion

Several structures of dimeric enolases of both prokaryotic [47] and eukaryotic [48,51] origin have been determined. By contrast, octameric arrangement has only been observed for enolases from *Streptococcus pneumoniae* [15] and *Streptococcus suis* [13]. Here we have determined the structure of the octameric enolase from *S*. *pyogenes* using X-ray crystallography. All streptococcal enolases exhibit some structural disorder in the L1 and L3 loop regions, as well as for the two C-terminal lysine residues. In our SEN^K312A^ structure, the L1 loop region shows two alternate conformations similar to the *S*. *pneumoniae* structure [15].

There is a range of prokaryotes, including GAS, that contain anchorless surface-associated glycolytic enzymes. It remains unknown as to how and why these enzymes are exported to the cell surface [13,52], although this is likely due to their multifunctional properties, which includes roles as virulence factors at the cell surface [53,54]. SEN has been proposed to contribute to the virulence of GAS by serving as a plasmin(ogen) receptor [6,7,55]. The main objective of our study was to use a combination of mutational analysis, three-dimensional structural analysis and biophysical approaches to uncover structure-function relationships relevant to the plasminogen-binding ability of streptococcal surface enolase. We targeted lysine residues for mutagenesis, because they typically play a key role in plasminogen binding. Unexpectedly, two mutant proteins with lysine-to-alanine substitutions (SEN^K312A^ and SEN^K362A^) bound plasminogen more strongly than observed for the wild-type protein. Both mutant proteins retained the alpha-helical fold and octameric structure of the wild-type protein. The stronger-binding mutant, SEN^K362A^, was shown to be less stable than wild-type SEN and SEN^K312A^. The CD spectra for SEN^K362A^ showed a slight decrease in the positive peak at 190–195 nm, which may indicate structural changes due to localized reorganization in the vicinity of the mutated residue. A similar trend was observed in thioredoxin mutants, where normally buried cysteine residues were modified [56]. Hence, changing a bulky amino-acid such as lysine to alanine can result in changes to the way secondary structural elements are packed.

Our structure shows that K362 is found to be located within the minor interface that is involved in the octamerization of the protein. The same lysine residue from *S*. *suis* is described to form a strong hydrogen-bonding network through close contacts with other residues that localize in the minor interface [13]. This cluster of interactions involving K362 is also present in our structure and its disruption may account for the increased plasminogen binding observed for SEN^K362A^. Although no significant difference is observed in the SEN^K362A^ structure in the vicinity of the substitution, this mutation is expected to make the octamer less stable, because both the inter- and intra-protomer interactions are affected. K312, on the other hand, is located on the outer edge of the octameric ring and is not involved in oligomerization. This may be the reason why this mutant behaves more like the wild-type protein.

Recently, there has been evidence to suggest that changes to residues involved in the minor interface result in not only a destabilization of the dimer-dimer interactions, but also weakening of the major interface [57]. The mass-spectrometry analysis presented here supports this theory, as SEN^K362A^ was shown to dissociate into monomers, with no indication of dimeric species being present. It has been suggested that one interface stabilizing the other may be important for the integrity of the functional unit [57,58]. Studies on dimeric enolases revealed that using different methods of dissociation to produce monomers resulted in variability of enzymatic activity, suggesting that the dimeric structure is necessary to stabilize the active site [57,59,60]. This may explain the reduction in glycolytic activity for the mutant proteins. Although the active site is completely contained within the monomer away from the octameric interfaces, a destabilization of these interfaces could change the integrity of the active site. In turn, this may also explain the increased propensity for SEN to bind plasminogen, as a loss of structural integrity may make the plasminogen-binding sites more accessible for the interaction with plasminogen. The sizes of the buried surface areas observed in the crystal likely reflect the stability of the octamer in solution, as the size generally correlates with the affinity of the interaction [61]. The K362A substitution in the subunit interface, and to a lesser extent, the K312A substitution outside the interface, appear to affect the stability of the octamer, which in turn can facilitate access of plasminogen to its binding sites.

We show that in the presence of SEN, the activation of plasminogen to form plasmin is enhanced. Once SEN binds plasminogen, we hypothesize that the interaction may induce a structural change in plasminogen, making it more susceptible to activation by tPA. The higher activation rate of plasminogen when mixed with SEN^K362A^ is likely to be due to an increase in the amount of plasminogen present in the tPA-susceptible conformation induced upon binding to SEN. Clearly, parameters additional to the stability of the SEN octamer can affect its ability to activate plasminogen, which may explain why we do not observe a better correlation between binding and activation in the three proteins compared here (Fig. 3).

In conclusion, we show that a change in the structural integrity of the SEN octamer, albeit small, has implications for the protein’s ability to bind and activate plasminogen. Little is known about the molecular basis of plasminogen binding to any of its receptors, and our study suggests on the one hand that the destabilization of SEN octamer facilitates access to plasminogen-binding sites, and on the other hand that SEN binding makes plasminogen more susceptible to tPA cleavage and activation. Our observations are in line with the recent suggestions that binding of SEN to a surface is critical for a formation of a stable complex with plasminogen [62]; such binding may facilitate access to plasminogen-binding sites by subtly destabilizing the interactions between protomers in octameric SEN, consistent with what we observe here. This conclusion is also entirely consistent with the limited accessibility to BS1 in the available bacterial SEN structures [13–15]. Our results advance our understanding of the mechanism of plasminogen binding by its receptors and have general implications for understanding the molecular basis of protein-protein interactions. However, further work is necessary to establish the details of the proposed mechanisms.
